# Major QTL with pleiotropic effects controlling time of leaf budburst and flowering-related traits in walnut (*Juglans regia* L.)

**DOI:** 10.1038/s41598-020-71809-x

**Published:** 2020-09-16

**Authors:** Şakir Burak Bükücü, Mehmet Sütyemez, Sina Kefayati, Aibibula Paizila, Abdulqader Jighly, Salih Kafkas

**Affiliations:** 1 Department of Horticulture, Faculty of Agriculture, University of Sütçü İmam, Kahramanmaraş, Turkey; 2 Department of Horticulture, Faculty of Agriculture, University of Çukurova, Sariçam, Adana, Turkey; 3 Agriculture Victoria, AgriBio, Centre for AgriBiosciences, Bundoora, VIC 3083 Australia; 4 School of Applied Systems Biology, La Trobe University, Bundoora, VIC 3083 Australia

**Keywords:** Plant sciences, Plant breeding

## Abstract

Breeding studies in walnut (*Juglans regia* L.) are usually time consuming due to the long juvenile period and therefore, this study aimed to determine markers associated with time of leaf budburst and flowering-related traits by performing a genome-wide association study (GWAS). We investigated genotypic variation and its association with time of leaf budburst and flowering-related traits in 188 walnut accessions. Phenotypic data was obtained from 13 different traits during 3 consecutive years. We used DArT-seq for genotyping with a total of 33,519 (14,761 SNP and 18,758 DArT) markers for genome-wide associations to identify marker underlying these traits. Significant correlations were determined among the 13 different traits. Linkage disequilibrium decayed very quickly in walnut in comparison with other plants. Sixteen quantitative trait loci (QTL) with major effects (*R*^2^ between 0.08 and 0.23) were found to be associated with a minimum of two phenotypic traits each. Of these QTL, QTL05 had the maximum number of associated traits (seven). Our study is GWAS for time of leaf budburst and flowering-related traits in *Juglans regia* L. and has a strong potential to efficiently implement the identified QTL in walnut breeding programs.

## Introduction

The genus *Juglans* consists of more than 20 species and is widely distributed worldwide^1^. *Juglans regia* L. is known as common walnut (English or Persian walnut), and is the most commercially important species in the genus due to its edible and highly nutritious nuts. Walnut is long-lived, deciduous, monoecious, open-pollinated and generally dichogamous^2^. *J. regia* is a diploid species with 16 haploid chromosomes^3^ and has an estimated genome size of ~ 606 Mb^4^. Genome of *J. regia* was previously reported by several authors^5–9^. Turkey has suitable ecological conditions for walnut cultivation and it produced about 5.5% (210,000 tons) of the total world production of 3,829,626 tons in 2017^10^.

Advanced molecular methods have revolutionized population genetics and evolutionary biology studies in different plants. The development of high-density DNA markers has played an important role in understanding the genetic diversity of different germplasm and in accelerating breeding programs and selection efficiency for complex traits in plants. Unfortunately, to date, there are few studies aiming to develop high throughput molecular marker systems to characterize walnut populations^11–17^. Walnut is a perennial species with a long juvenile period resulting in the time-consuming breeding cycle. Walnut breeding would therefore greatly benefit from the development of molecular markers that could be used for diversity assessment, gene discovery, marker-assisted selection, genomic selection, and other breeding applications. Over the past two decades, single nucleotide polymorphism (SNP) markers have become the ultimate choice to characterize germplasm due to the possibility of developing thousands of SNPs in a considerably short time using next-generation sequencing technologies (NGS).

Genotyping by sequencing (GBS) has been developed to make simultaneous SNP discovery and genotyping as a rapid and robust method for reduced-representation sequencing of multiplexed samples that combines genome-wide molecular marker discovery and genotyping^18^. The Diversity Arrays Technology (DArT) is a cost-effective sequence-independent ultra-high-throughput marker system. This technology has developed a GBS platform known as DArT-seq, which provides an opportunity to select genome fractions corresponding predominantly to active genes. Restriction enzymes used in this method separate low copy sequences that informative for marker discovery from the repetitive fraction of the genome. Then, representative fragments are sequenced on Next Generation Sequencing (NGS) platforms. As a result, DArTseq offers affordable genome profiling by producing high-density SNPs as well as markers of presence and absence^19^.

In plant breeding, one of the main aims is to determine the genetic dissection of quantitative traits. In this regard, quantitative trait loci (QTL) that are affecting the observed phenotype can be detected by using linkage mapping and linkage disequilibrium analysis. Linkage mapping is restricted to a relatively low genomic resolution when evaluating the recombination events within the mapping populations. An alternative is genome-wide association mapping (GWAS) that exploits linkage disequilibrium (LD) in diverse populations to find putative QTL. This method has several advantages over linkage mapping as it uses natural variation, and has higher genetic resolution due to exploiting historical recombinations^20^. Recently, GWAS was also applied to walnut for ecophysiological traits such as water use efficiency as estimated by carbon isotope discrimination, and photosynthetic capacity^17,21^ as well as phenology, yield and lateral bearing traits^14,15^; while several other studies reported a few QTLs associated with nut-related traits^12,16^. However, QTL studies in walnuts are limited when it compared to other plant species.

For perennial fruit species, in the context of global warming, endodormancy release may be a critical step in the future due to insufficient chill accumulation, directly affecting flowering quality and uniformity, and thus leading to a drastic reduction of fruit production^22^. Walnut tree, like the other decidious tree species, requires winter chilling and heat the breaking of dormancy in the spring^23^. On the other hand, time of budburst and leafing date traits are very important in walnut cultivation due to spring frost that may cause significant losses in the production in some years especially in Turkey where it is prevalent^24^. Flowering-related traits are key factors not only in the plant life cycle but also in determining the productivity for walnut. The female flower buds can bear at the terminal and lateral shoots of walnut depending on cultivar. Particularly, the lateral bud flowering, nut setting types and female flower abundance are one of the most important features in walnut breeding programs due to association with high yield^24,25^. In addition, syncronizing the flowering habit of males and females is another important issue in terms of fertilization biology in a walnut orchard. Furthermore, high heritability were indicated for leafing date, heterodichogamy and female/male blooming were in walnut^14,26^. Therefore, the development of molecular markers related to phenology and flowering traits in walnut will play an important role in future breeding programs and will facilitate the development of other morphological, nutritional and physiological characteristics without being confounded by flowering time differences among individuals.

In this study, we have investigated the walnut population showing significant variations in terms of leaf budburst and flowering-related traits located in the Kahramanmaraş province, which is one of the walnut growing areas in Turkey, to (1) understand the genetic diversity and structure of walnut population and study its linkage disequilibrium, and (2) identify associations between molecular markers with time of leaf budburst and flowering-related traits using genome-wide association mapping to aid marker-assisted breeding in walnut. Besides, this study is the first study to identify markers associated with several yield-related traits such as catkin abundance, female flower abundance, nut setting type, and inflorescence habit in walnut.

## Materials and methods

### Plant materials

The walnut collection and breeding studies carried out in the Nuts Application and Research Center (SEKAMER), Kahramanmaraş, Turkey. The SEKAMER is located at 37° 35′ 27ʺ N latitude, 37° 03′ 28ʺ E longitude and 930 m above the sea level. Kahramanmaraş has a mild climate between the Mediterranean and continental with 727 mm yearly precipitation and 16.9 °C average yearly temperature. In this study, a collection of 188 walnut accessions (Supplementary Table S1) in SEKAMER were used for association mapping. The 188 walnut accessions were selected from approximately 1,200 accessions in the germplasm based on their difference from each other in terms of their phenology, yield and nut related traits as well as their age. The accessions were at 9–12 ages during this study.

### Phenotyping

A total of 13 phenological traits were observed during the budburst and flowering period in the 2016, 2017 and 2018 growing seasons for 188 walnut accessions. The accessions were characterized based on the Descriptor for Walnut^27^ and the International Union for the Protection of New Varieties of Plants^28^. A list of characteristics and their definitions were presented in Table 1. The observation of phenotypic data as dates was recorded as the number of Julian days from January 1st of each year.

**Table 1:** Codes and definitions used in the determination of phenological traits.

Codes	Traits	Description
T01	Time of leaf budburst	When over 50% of terminal buds have enlarged and the bud scales have split exposing the green of the leaves inside
T02	Leafing time	Date when 50% of terminal buds have enlarged and the bud scales have split exposing the green leaves
T03	First female blooming time	Date of initial pistillate flower receptivity
T04	First male blooming time	When first pollen shedding occurs
T05	Last female blooming time	Date of last pistillate flower receptivity
T06	Last male blooming date	When last pollen shedding occurs
T07	Blooming period of female flowers	Receptive period of female flowers
T08	Blooming period of male flowers	Receptive period of catkins
T09	Catkin abundance	3 low; 5 intermediate; 7 high
T10	Female flower abundance	3 low; 5 intermediate; 7 high
T11	Nut setting type	1 Solitary; 2 Binate; 3 Fascicled; 4 Bunchy
T12	Inflorescence habit	1 Protandrous; 2 Protogynous; 3 Homogamous
T13	Lateral bud flowering (lateral fruitfulness)	Percentage of lateral shoots with female flowers

Source: IPGRI^27^; UPOV^28^.

### DNA extraction and genotyping

Collected leaves were washed with distilled water in order to clean dust, frozen in liquid nitrogen, and stored at − 80 °C until DNA extractions. Genomic DNA extraction was carried out according to the procedure described^29^ with minor modifications^30^. The purity and quantity of extracted DNA were measured using agarose gel electrophoresis and Qubit fluorometer (Invitrogen) according to the manufacturer's protocol. DNA concentrations of all accessions were fixed to 80–100 ng/µl for DArT-seq analysis.

DNA samples were processed in digestion/ligation reactions for complexity reduction using *Pst*I-*Mse*I as described^31^ to generate the DArT-seq markers. DArTSeq markers with MAF < 5% and missing data > 20% were discarded from all analyses. The physical positions of the DArT-seq markers were obtained by aligning marker sequences on the walnut reference genome^5^. BLAST^+^ software (https://www.ncbi.nlm.nih.gov/bookshelf/br.fcgi?book=helpblast) was used to adentify the physical positions of the markers with word size equal to 20 and evalue < 1e − 10. For sequences with multiple hits on the genome, the hit with the smallest evalue was considered as the physical position.

### Statistical analysis

Pairwise correlations between different phenotypes were calculated using R (https://www.r-project.org). The hierarchal clustering of phenotypes and individuals using the phenotypic data was calculated using Euclidean distance and was plotted using the R function ‘heatmap.2’. SNPs with known scaffold positions were used to estimate the LD between markers using the squared allele frequency correlation (*R*^2^) following Hill and Weir^32^. Pairwise *R*^2^ values among SNPs located on the same scaffold were plotted against the physical distance (bp) between both SNPs to estimate the LD decay. The second-degree loess smoothing was calculated and plotted using R. Population structure was defined using the software ADMIXTURE^33^. The analysis was run with k value ranging from 2 to 20 and ten cross validations^33^. To avoid bias due to the linkage disequilibrium, we used PLINK^34^ software to prune SNPs at *r*^2^ value of 0.5. We ran 100 replicates of the ADMIXTURE analysis for each *k*. The most probable *k* was defined as the smallest *k* value in which the cross validation values of the hundred replicates had no significant difference from that for the next *k*.

### Genome-wide association study

Restricted maximum likelihood (REML) analysis was used to calculate the narrow sense heritability by fitting the genotypic and phenotypic data as described in Yang et al.^35^. The mixed linear model (MLM) implemented in the genome association and prediction integrated tool (GAPIT) R package was used for the GWAS analysis^36^. The same package calculates the principal component (PC) analysis and the genomic relatedness matrix using VanRaden^37^ method and fits them as covariates for the GWAS analysis. The Bayesian information criterion (BIC) was used to find the optimal number of PCs to be fitted in the GWAS analysis for each trait. Bonferroni^38^ test was used to declare significant threshold at *p* < 0.05 for the GWAS analysis. Markers detected at this threshold were declared as highly significant markers. However, because of the stringency of Bonferroni test that ignores linkage disequilibrium and assumes independent markers, we reported all DArT-seq markers with *p* < 10^−4^ as suggestive associations if they had high linkage disequilibrium with a highly significant marker associated with a multi-trait QTL (defined when a marker is associated with several different traits). The full scaffold ordering information was obtained from Kefayati^39^ in which they used an F1 population with 175 individuals from a cross between Chandler and Kaplan-86 walnut cultivars to develop a genetic map. Predicted genes were extracted from the genome if they had a distance smaller than 20 kb from the associated marker. This value was selected based on the linkage disequilibium results.

## Results

### Phenotypic variations and correlation between phenotypic traits

In the present study, the data for 13 phenological traits related to leaf budburst and flowering were collected during three growing seasons (2016, 2017 and 2018). Phenological data related to leaf budburst and flowering traits are presented in Supplementary Table S2. Significant variations were obtained in most of the studied phenological traits. The budbreak date ranged from 67 (2018) to 109 (2017) days, while leafing time ranged from 76 (2018) to 117 (2017) days. While the first and last female blooming dates were 104.96 and 117.51 Julian days, the first and last male blooming dates were 102.05 and 109.03 Julian days on average, respectively. Female flowering period of walnut accessions varied from 4 to 25 days, and male flowering period varied between 2 and 15 days during three years. Female abundance, fruit setting type, and lateral bud flowering are important yield parameters in walnut. Walnut accessions were approximately ‘moderate’ in terms of female flower abundance (5.65) and catkin abundance (4.78) on average. Identification of ‘solitary’ and ‘bunchy’ walnut genotypes in all vegetation periods was an important source of variation for fruit setting type. Each walnut accessions had different inflorescence habits, while ‘protandrous’ was predominant. The average percentage of lateral bud flowering was 64.7%. While seasonal shifts in phenological findings were observed between the years, consistent findings were obtained between walnut accessions during three consecutive years. In addition, the estimation of narrow-sense heritabilities ranged from 12.3 to 95.4% for the average of traits across the three growing seasons suggesting that the considerable proportion of the phenotypic variation for most of the evaluated traits can be explained by genetic factors. High narrow-sense heritabilities (*h*^2^) were obtained for the budburst (0.877) and leafing times (0.888) as well as for the first (0.936 and 0.715) and the last (0.846 and 0.611) female and male blooming dates, respectively. The *h*^2^ values for inflorescence habit (0.552), lateral bud flowering (0.538) and female abundance were intermediate (0.455), while the values for female (0.123) and male (0.136) flowering periods, catkin abundance (0.188) and nut setting type (0.147) were low.

**Figure 1:**
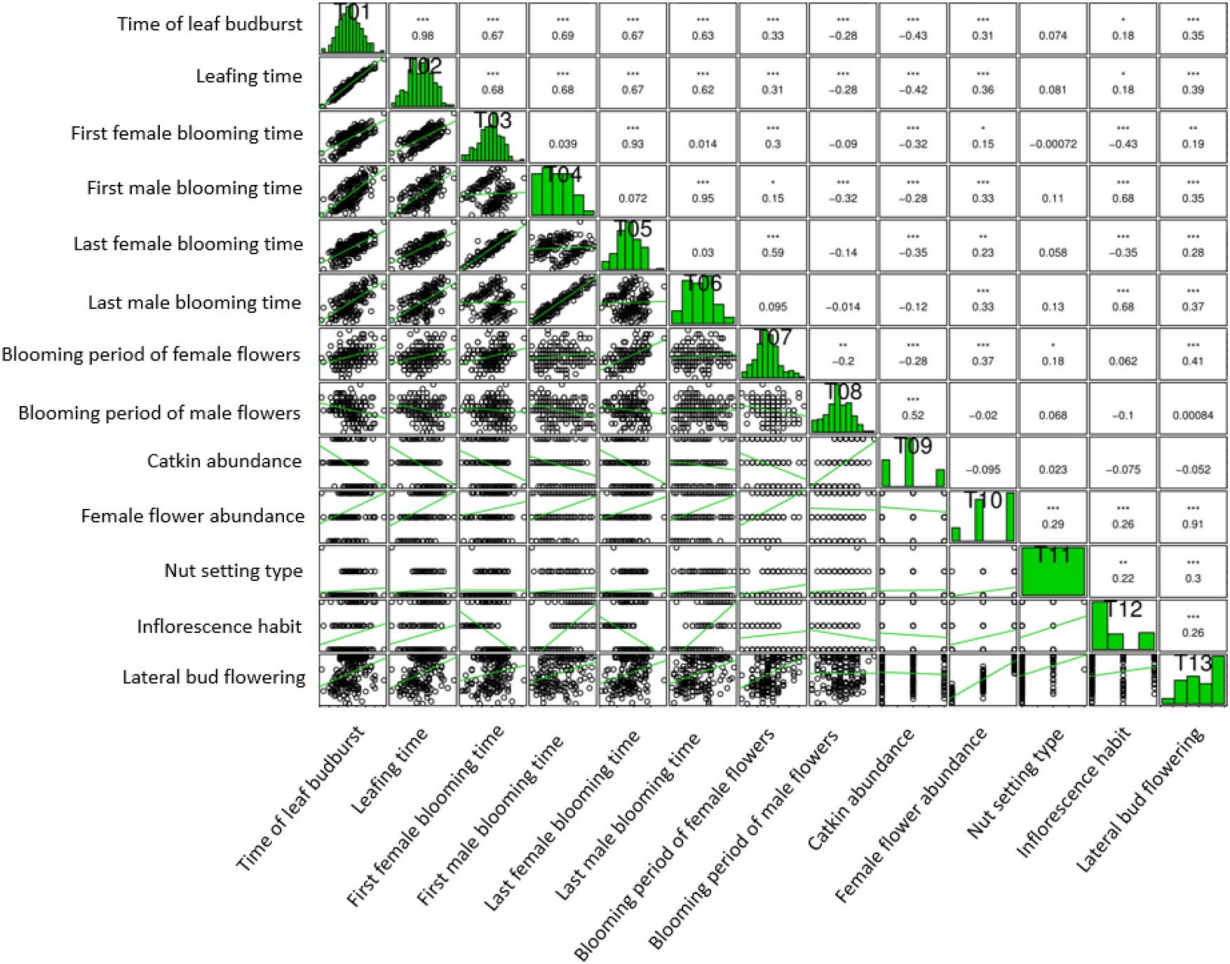
Correlation analysis results of phenological characteristics related to time of leaf budburst and flowering-related traits.

Many traits related to flowering and time of leaf budburst showed significant correlations (Fig. 1; Supplementary Data 1). Highly significant positive correlations were observed between budburst and leafing times with other traits, except nut setting type and female and male flowering periods, but both traits were negatively correlated with male flowering period and catkin abundance. The first and the last female blooming dates had positive correlations with female flowering period (0.30 and 0.59), while they had significantly negative correlations with catkin abundance (− 0.32 and − 0.35) and inflorescence habit (− 0.43 and − 0.35) at *p* = 0.001. The first and the last male blooming dates were correlated positively with female abundance (0.33 and 0.33), inflorescence habit (0.68 and 0.68) and lateral bud flowering traits (0.35 and 0.37), while the first male blooming dates had negative correlations with catkin abundance (− 0.28) and male flowering period (− 0.32) at *p* = 0.001. Female flowering period correlated significantly and positively with female abundance (0.37) and lateral bud flowering traits (0.41) at *p* = 0.001. However, it was correlated negatively with catkin abundance at *p* = 0.001 (− 0.28) and male flowering period at *p* = 0.01 (− 0.20). There was a positive correlation between male flowering period and catkin abundance (0.52) at *p* = 0.001. Female abundance and lateral bud flowering traits had significant positive correlations with all studied traits except male flowering period and catkin abundance. The nut setting type was positively correlated with female flower abundance (0.29) and lateral bud flowering (0.30) at *p* = 0.001. In addition to these results, inflorescence habit was positively correlated with lateral bud flowering at *p* = 0.001 (0.26).

### Phenotypic and genotypic diversity and linkage disequilibrium in the walnut germplasm

**Figure 2:**
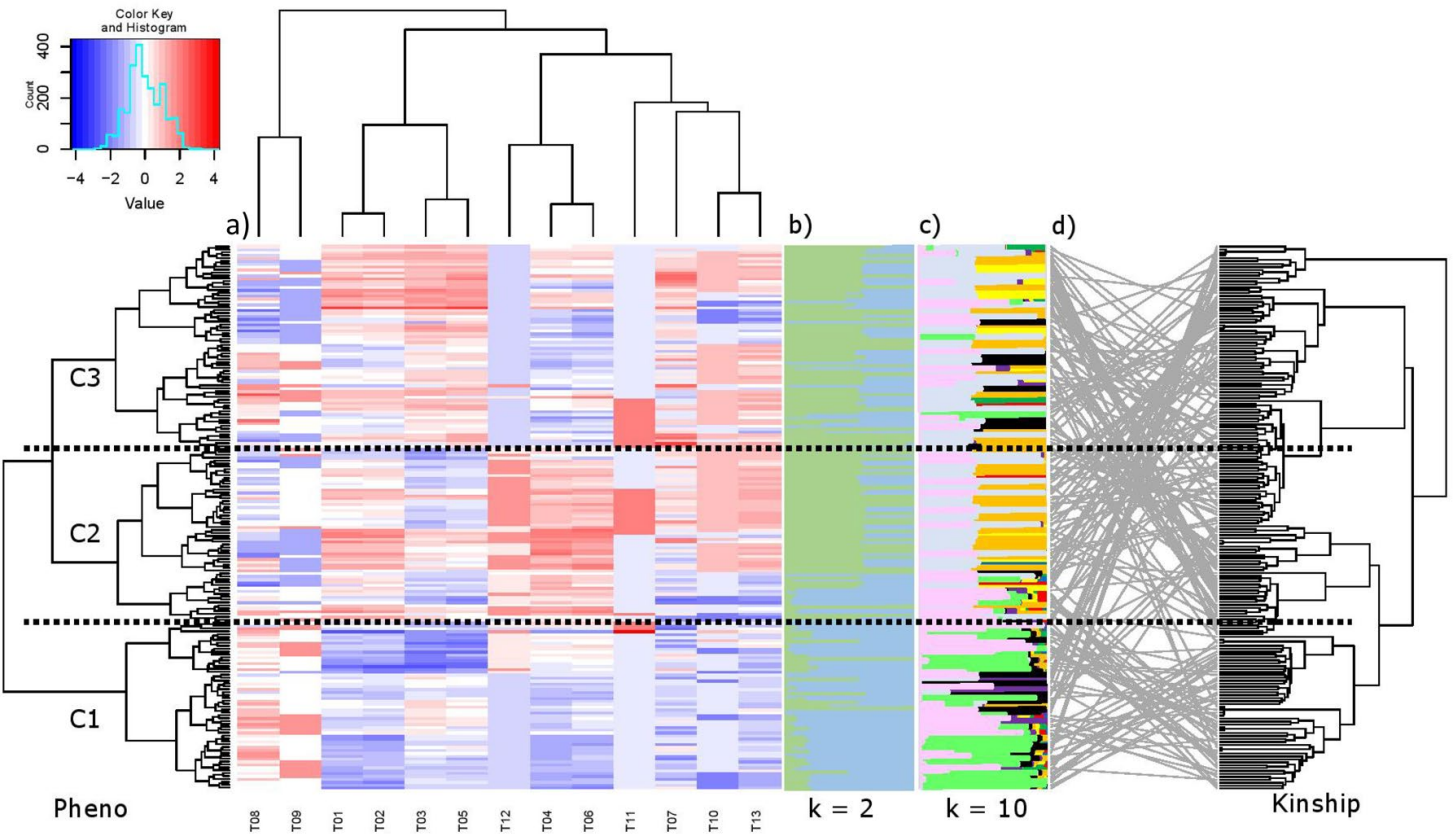
(**a**) Phenotypic clustering among 13 phenological characteristics reletad to flowering measured and population sutrucutere in 188 walnut genotypes; (**b**) population structure results for k = 2; (**c**) population structure results for k = 10; (**d**) clustering among genotypes based on the kinship relatedness matrix and their relation to the phenotype-based clustering.

The 13 phenotypic traits were grouped into eight clusters. The first one includes flowering period of male flowers and catkin abundance, while the second one contains time of leaf budburst and leafing time. The first and the last blooming dates of female flowers were in the third cluster, while inflorescence habit was in the fourth cluster. The first and the last blooming dates of male flowers were in the fifth cluster, while nut seting type and flowering period of female flowers were in sixth and seventh clusters, respectively. The last cluster included female flower abundance and lateral bud flowering traits (Fig. 2a). Clustering the germplasm based on the 13 phenotypic traits divided it into three distinguished clusters (Fig. 2a). Cluster one (C1) generally had phenotypic values below (blue colour in Fig. 2a) the average for all traits except male flowering period (7.7 vs 6.8 for C2 and C3) and catkin abundance (5.3 vs 4.4 for C2 and C3) in which it showed above average phenotypic response (red colour in Fig. 2a). Cluster two and cluster three (C2 and C3) are more similar to each other than cluster one. For C2 and C3, above average phenotypic values were observed for the traits time of budburst (88.3 vs 84.3 for C1), leafing time (98 vs 94.2 for C1), female flowering period (12.9 vs 11.7 for C1), female abundance (6.1 vs 4.9 for C1) and lateral bud flowering (74 vs 49 for C1); and values were below average for male flowering period and catkin abundance, and variable responses for nut setting type. However, C2 had phenotypic values above the average for traits the first and the last male blooming dates and inflorescence habit and values below the average for the first and the last female blooming dates were in contrast to C3 (Fig. 2a).

In this study, a total of 23,029 DArT-seq derived SNPs and 30,031 DArT markers were identified, and were reduced to a total of 33,519 (14,761 SNP and 18,758 DArT) markers after filtering out markers with low allele frequency (< 5%) and high missing data (> 20%). Population structure analysis showed that 10 (Fig. 2c) could be a reasonable estimation of the number of ancestral subpopulations as the CV values for the 100 replicates of *k* = 10 was not significantly different from that for *k* = 11. PCA analysis also confirmed the presence of complex structure in our gemrplasm with multiple clusters distributed along the first three PCs, which explained 10.4%, 6.3% and 4.4% of the total variation, respectively (Supplementary Fig. S1). However, at *k* = 2 (Fig. 2b), the majority of the phenotypic C1 cluster formed one subpopulation with considerable admixture with the other subpopulation. Almost 82.6% of C1 was from Q1 (blue) and 17.4% from Q2 (green), while C2 and C3 were 36.2% from Q1 and 63.8% from Q2. Similarly, the dendrogram based on the kinship relatedness isolated large proportion of the phenotypic C1 in one group (Fig. 2d; Supplementary Fig. S2).

The walnut germplasm showed rapid LD decay with small LD blocks indicating very large ancestral effective population size (Supplementary Fig. S3). Using SNPs that exists on the same contig, LD decayed below *r*^2^ value of 0.2 at 16.6 kb and below *r*^2^ value of 0.15 at 41.2 kb. For this germplasm, our marker spacing was approximately 18.1 kb (~ 606 Mb genome size divided by 33,519 markers). For this reason, it seems that we achieved sufficient marker coverage in our germplasm.

### Genome-wide association study

**Figure 3:**
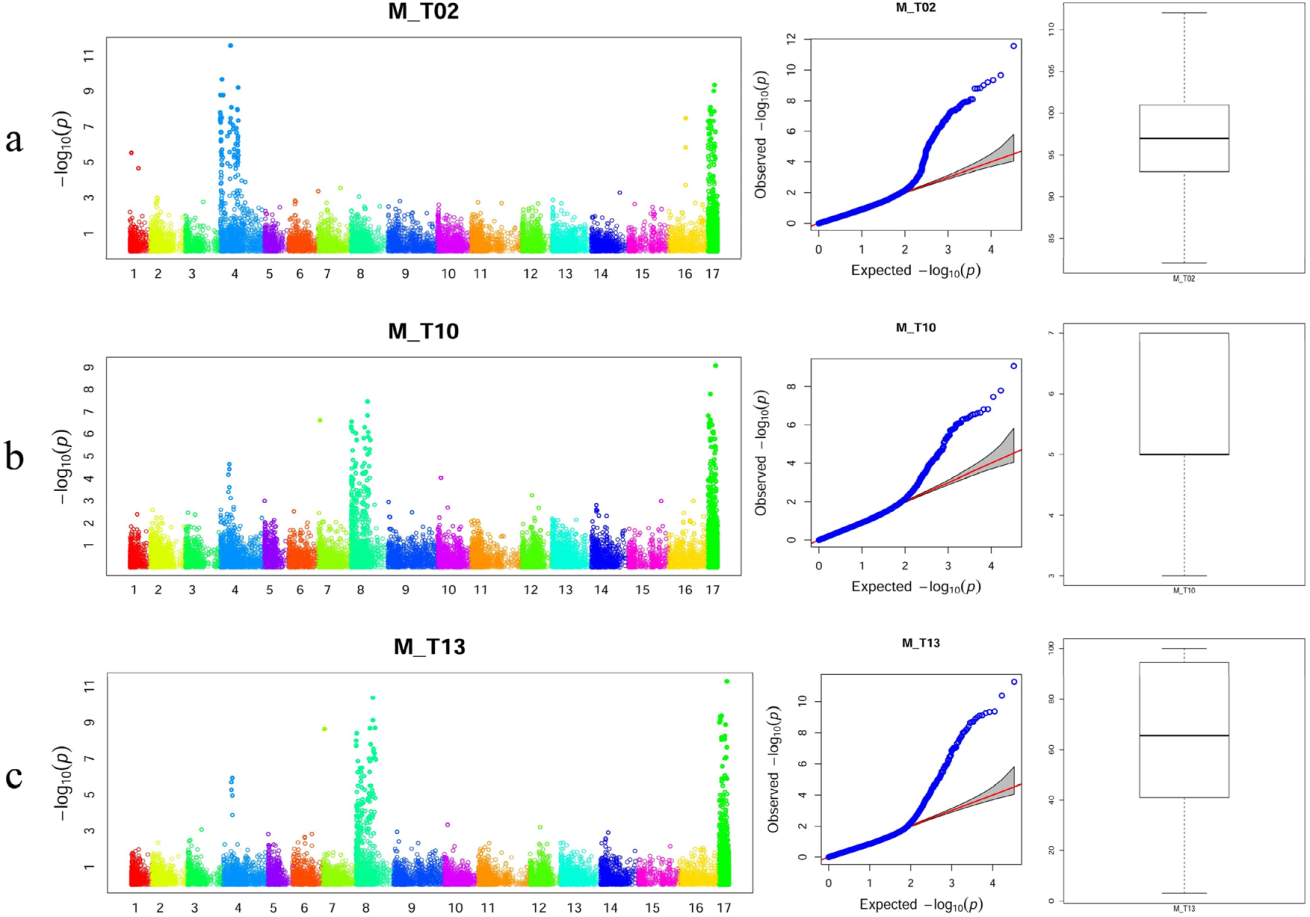
Manhattan plots, Quantile–quantile plots and Boxplots from association analysis of three phenological traits; (**a**) leafing time, (**b**) female flower abundance, (**c**) lateral bud flowering.

The significant threshold for the GWAS analysis was equal to 1.5 × 10^−6^ (0.05/33,519) following the Bonferroni method. Two hundred and forty-six unique markers showed a total of 1,285 significant marker trait associations with time of leaf budburst and flowering-related phenotypes across all traits and seasons (Supplementary Data 2). These 1,285 associations were clustered in 16 multi-trait QTL distributed on eight different LGs, of which one had unknown genomic positions (Table 2). LG8 had the highest number of QTL (six) followed by LG4 with three different QTL. The *R*^2^ values for all the sixteen QTL across different traits and seasons ranged between 0.08 and 0.23 indicating the presence of major QTL controlling different traits (Fig. 3; Table 2; Supplementary Data 2). Within 20 kb flanking the highly associated markers, we detected a number of flowering related domains such as *ESCAROLA, FERONIA, TCP20, FERONIA* and *MYB* for all QTL in general (Supplementary Data 2).

**Table 2:** In walnuts, significant QTL associated with time of leaf budburst and flowering characters.

QTL	Traits	Seasons	Best marker	LG	Pos	Best P	MAF	Best *R*^*2*^
QTL01	1, 2, 6	All	100044119|F|0–5:A>C–5:A>C	1	860016	1.5E − 06	0.31	0.08
QTL02	4, 6, 12	18, M	7409645|F|0–50:A>T–50:A>T	3	16331345	4.88E − 06	0.49	0.11
QTL03	1, 2, 3, 6	All	7405671|F|0–60:T>G–60:T>G	4	4553313	6.25E − 10	0.33	0.12
QTL04	10, 13	16, 17, M	12416095|F|0–51:G>A–51:G>A	4	20274583	5.18E − 07	0.20	0.13
QTL05	1, 2, 3, 4, 5, 6, 11	All	100010320|F|0–47:C>T–47:C>T	4	28418795	2.71E − 12	0.26	0.16
QTL06	10, 13	All	7397430|F|0–16:G>A–16:G>A	7	437368	2.3E − 09	0.27	0.17
QTL07	3, 4, 5, 6, 12	All	7397034	8	3779475	2.49E − 09	0.23	0.2
QTL08	3, 4, 6, 12	17, 18, M	100033978|F|0–67:A>G–67:A>G	8	4413427	9.9E − 07	0.23	0.12
QTL09	10, 13	16, 17, M	7402360|F|0–17:C>G–17:C>G	8	12387115	1.80E − 09	0.23	0.19
QTL10	8, 10, 13	All	12413277	8	14505338	5.14E − 12	0.35	0.23
QTL11	10, 13	All	12416675|F|0–66:T>A–66:T>A	8	21210755	2.8E − 08	0.18	0.15
QTL12	10, 13	16, 17, M	7398458|F|0–28:T>A–28:T>A	8	23526767	5.7E − 07	0.17	0.12
QTL13	3, 4, 5, 6, 12	17, 18, M	23446637|F|0–32:T>C–32:T>C	11	19125490	3.15E − 07	0.14	0.15
QTL14	4, 6, 12	17, 18, M	100050947	12	12458779	7.5E − 09	0.12	0.18
QTL15	1, 2, 4, 6	All	23447831|F|0–31:T>G–31:T>G	16	2561937	2.19E − 08	0.28	0.11
QTL16	10, 13	16, 17, M	7406005	NA	NA	8.34E − 11	0.45	0.22

Markers were clustered into QTL based on their LD and the marker with the lowest-value across traits and seasons were reported here. Detailed informations can be found in Supplementary Data 2.

QTL05 was associated with seven different traits (budburst, leafing, first and last blooming dates for male and female as well as nut setting type) which is the largest number compared to other QTL, with *R*^*2*^ values between 6 and 16%. The allelic effect ranged from 2 to 5 days for the first six traits and 0.18 points on the scale of nut setting type (Supplementary Data 2). There were seven QTL controlling both female abundance and lateral bud flowering distributed on LG4, LG7 and LG8, as well as one QTL with unknown position. The best *R*^*2*^ values for these QTL ranged between 9 and 18% for female abundance and 12–23% for lateral bud flowering and the allelic substitution effect ranged from 0.84 to 1.3, and 17.5–30%; respectively (Supplementary Data 2). One of these QTL, QTL10, was also associated with male flowering period explaining 11% from its variation and having an effect of one day.

QTL02, QTL08 and QTL14 were all associated with first and last male bloom data and inflorescence habit explaning from 6 to 11%, 7–12% and 8–18% for each of the phenotypic variation for the three traits; respectively. The allelic substitution effects ranged from 2.8 to 4.9 days for male blooming and 0.4–1 for inflorescence habit. QTL08 was also associated with first female blooming date with *R*^2^ value of 6% and allelic effect of 3.1 days. QTL07 and QTL13 were associated with the first and last blooming dates for male and female (*R*^2^ between 6 and 14% and effect between 3.9 and 8.6 days) as well as inflorescence habit with *R*^2^ = 20% and effect = 1.3 for QTL07 and *R*^2^ = 15% and effect = 1.2 for QTL13. The remaining QTL (QTL01, QTL03 and QTL15) were associated with budburst, leafing and last make blooming dates, explaining from 8 to 12%, 6–11% and 6–8% for each of the three traits; respectively. QTL03 was also associated with first female blooming date while QTL15 was assocaited with first female blooming date. The allelic effects ranged between 2.2 and 5.1 days.

## Discussion

### Phenotypic data and genetic diversity

Time of leaf budburst and flowering habits are complex phenological traits that are influenced by a variety of physiological and environmental factors and they vary widely among genotypes. Genetic and agronomic improvements have greatly increased the yield potential of plants. Therefore, it is very important to associate these traits genetically and to establish the correlation between them^40^. In the present study, significant phenotypic variations were detected for 13 important phenological characteristics related to time of leaf budburst and flowering-related traits among walnut accessions. We obtained high narrow-sense heritability values for budburst and leafing dates as well as for the first and the last female and male flowering dates, while the values for female and male flowering periods were low as reported previously^14,15^. The narrow-sense heritability value for lateral bud flowering (0.538) was intermadiate in thsi study, while Marrano et al*.*^14^ obtained very high value (0.98).

In this study, positive correlations were detected between time of budburst and female-male blooming dates with similar findings by Bernard et al*.*^15^, while there were no correlations between female and male blooming dates as previously indicated^15^. Lateral bud flowering is one of yield parameters in walnut, and a negative correlation (− 0.24) was reported^14^ between leafing date and lateral bud flowering, while a positive correlation (0.39) was found in this study. This can be explained by different populations used in two studies. Long flowering period is an important trait due to get high percentage of nut settings. Male flowering period was usually shorter than female ones in this study as reported previously^15^. Catkin abundance was found as a strong indicator for long male flowering period due to high correlation between them. In this study, female flowering period and female flower abundance had positive correlations with most of the traits, while male flowering period and catkin abundance had negative ones. Low correlations and low istatistical significances were indicated between female-male flowering period and other traits^15^.

Choosing the right populations is critical for successful association mapping. The selected natural walnut population must have a sufficiently high genetic diversity. Our walnut accessions consisted of 188 walnut genoypes which are common cultivars in USA and in France as well as some important domestic Turkish walnut cultivars and genotypes that have outstanding characteristics. Therefore, our population had a very wide genetic diversity. Genetic diversity of different *J. regia* L. germplasm has previously been described in various studies^12–17,41–48^ and it was shown that walnut genotypes conserved a high level of genetic diversity (Fig. 2; Supplementary Fig. S2). The kinship (Supplementary Fig. S2) analyses confirmed the presence of massive diversity in this germplasm. Moreover, LD decayed quickly at around 20 kb indicated large effective population size for the walnut population.

Previous population genetic structure studies in *J. regia* used SSR markers and focused on germplasms distributed on restricted geographical regions with limited diversity such as Indian genotypes^46^, trees from cold temperate areas of the USA and Europe^44^, and Chinese genotypes^43,48^. Another study investigated the natural population of *J. regia* in the eastern Italian Alps where moderate genetic diversity was found^45^. Using the Axiom 700 K SNP array, Arab et al.^12^ divided a set of 95 Iranian accessions into four subpopulations. The same SNP array was used by Bernard et al*.*^15^, and the genotypes originating from Western Europe and America were located in one cluster and the genotypes of Eastern Europe and Asia origin were in the other cluster, while French, American and native walnut genotypes were not sharply separated in this study. Erman et al*.*^16^ used a total of 13,611 DArT derived SNP markers, and two peaks were detected at K = 3 and K = 5 in the cluster analysis of 154 walnut accessions, which did not cluster according to their geographical origin. The walnut cultivars and outstanding genotypes were included in this study as the analysis revealed the existence of ten ancestral subpopulations. Even though many genotypes with similar phenotypes were genetically clustered together, the ADMIXTURE analysis at *k* = 2 showed considerable mixing between early and late blooming accessions.

### GWAS

Time of leaf budburst is a very important trait for fruit trees because of the importance of identifying earliness or lateness and mechanisms for their adaptability to different ecological conditions. There have been few association mapping studies on budburst and leafing time for tree species. Flowering-related traits are usually considered as important indicators for productivity. Association mapping studies have been carried out in different plants related to flowering traits such as rice^49,50^ and apple^51^. In wheat, barley, and soybean, for example, major genes controlling flowering time were reported to be associated with yield in different climates^52–54^. In walnut, Kefayati et al.^55^ identified a major QTL for leafing time using a ‘Chandler × Kaplan-86’ F1 segregating population. Similarly, a complex genetic archtictue for flowering related traits in a diverse germplasm was also observed in walnut by Bernard et al.^15^ with multiple major and minor genes contributing to the variation of different traits. Marrano et al.^14^ also detected several QTL associated with leafing and harvest dates. In the present study, we detected the first markers associated with some characteristics such as catkin abundace, female flower abundance and nut setting type, which are very important for yield-related traits in walnut. Besides, we revealed that several highly significant markers associated with major multi-trait QTL for different phenological traits. In general and as expected, almost each detected QTL was associated with multiple significantly correlated traits.

A number of the QTL reported in this study overlapped with or were flanked by genes with annotations that are known to play critical roles in flowering time in other plant species. QTL01 is only 3.4 kb away from the gene *WALNUT_00020424* which is related to *FAR1* (Supplementary Datas 1 and 2). *FAR1* is a transcription factor that regulates the phytochrome A signaling pathway in *Arabidopsis thaliana* and regulates the gene *ELF4* that is essential to maintain the circadian rhythm^56,57^. The most significant SNP associated with QTL08 (*100033978|F|0–67:A*>*G–67:A*>*G*) is located within the gene *WALNUT_00018132* that encoded a BEACH domain-containing protein. The BEACH domain was previously reported to be associated with flowering and flower organ development in Arabidopsis^58^. Multiple markers on the scaffold *jcf7180001222160* showed highly significant associations with QTL10. Of those, the markers *12413277* and *23443558* showed highly significant association with male flowering time, female abundance and lateral bud flowering in all seasons. These markers were linked to the genes *WALNUT_00006818* and *WALNUT_00006828* which encoded DNA-binding protein *ESCAROLA* and auxin efflux carrier component 8 (*PIN8*), respectively (Supplementary Data 2). *ESCAROLA* was previousuly reported to be responsible for modulating hypocotyl growth inhibition in response to light in Arabidopsis^59^ and affecting leaf senescence^60,61^ as well as flowering time^62^. *PIN8* was previously reported to be involved in regulating auxin dependent transcriptional activity which has a major effect on the growth of lateral buds^63^. Thus, it seems that QTL10 is associated with multiple genes that are controlling flowering and leafing traits.

QTL05 is another example of QTL that seems to be associated with multiple genes. This QTL had the largest number of highly significant marker-trait associations that are located on different scaffolds with high LD with each other (Supplementary Data 2). Seven of these markers overlapped with or flanked genes with flowering and leafing related annotations. These annotations included *FERONIA*, *TCP20*, auxin-induced, *ankyrin* and MYB related proteins. *FERONIA* is known to play a critical role in female control of pollen tube, growth, development, hormon signalling and stress tolerance in different organisms^64^; while *TCP20* was previously reported to modulate flowering^65^. McClure^66^ reported an SNP associated with flowering time in the apple that is linked to an *ankyrin* repeat-containing protein *At2g01680*-like, which is the same annotation for the walnut gene *WALNUT_00004309* that overlapped with the marker *7394079* in this study. Three markers also associated with QTL05 were linked to MYB related genes which are *WALNUT_00010727*, *WALNUT_00028043,* and *WALNUT_00031245*. MYB is a large gene family that has been extensively studied and many members of this family were previously reported to affect anther development, flowering time and to get upregulated in flowers in different plants including *Arabidopsis thaliana*, rice, and bamboo^67–70^. QTL03 was also linked to the MYB related gene *WALNUT_00008342* (Supplementary Data 2).

## Conclusion

In summary, our study confirmed that time of leaf budburst, leafing time and flowering-related characteristics are complex phenotypic traits. In this study, a large amount of phenotypic variation was observed for the phenological traits studied and presented an important opportunity to examine the genetic architecture of these complex traits. This is the first GWAS study in walnut providing results from high number (13) of traits studied together, and results for the first time from several traits such as catkin and female flower abundance, nut setting type, and inflorescence habit. Using GWAS, we identified a large number of loci and significant 16 QTL associated with leaf budburst time and flowering-related traits. These genes can be used in marker-based breeding programs for earliness/lateness and productivity in the walnut. Moreover, the high resolution of linkage disequilibrium mapping in this study will facilitate the determination of the exact causal variants and underlying genes controlling these traits in future studies. Thus, this study is a step toward understanding the molecular pathway involved in the flowering response in walnut. Future research should focus on the effect of combining different alleles of these genes to characterize their impact on flowering time. Controlling the time of flowering is one of the main factors affecting walnut yield, thus, ensuring flowering at the optimal time will avoid yield loss. The markers detected in this research can facilitate the selection of walnut trees harboring favorable combinations of flowering genes to optimize its timing.

## Supplementary information

Supplementary Data 1.

Supplementary Data 2.

Supplementary Figures.

Supplementary Tables.
