# Supplementary material for: Major QTL with pleiotropic effects controlling time of leaf budburst and flowering-related traits in walnut (Juglans regia L.)
Source: Sci Rep. 2020 Sep 16;10:15207. doi: 10.1038/s41598-020-71809-x (PMC7495441; doi:10.1038/s41598-020-71809-x)
Supplement: Supplementary file 3 — Supplementary Figures. [file 41598_2020_71809_MOESM3_ESM.docx]

Supplementary Figures

**Major QTL with pleiotropic effects controlling time of leaf budburst and flowering-related traits in walnut (*Juglans regia* L.)**

Şakir Burak Bükücü, Mehmet Sütyemez, Sina Kefayati, Aibibula Paizila, Abdulqader Jighly, Salih Kafkas


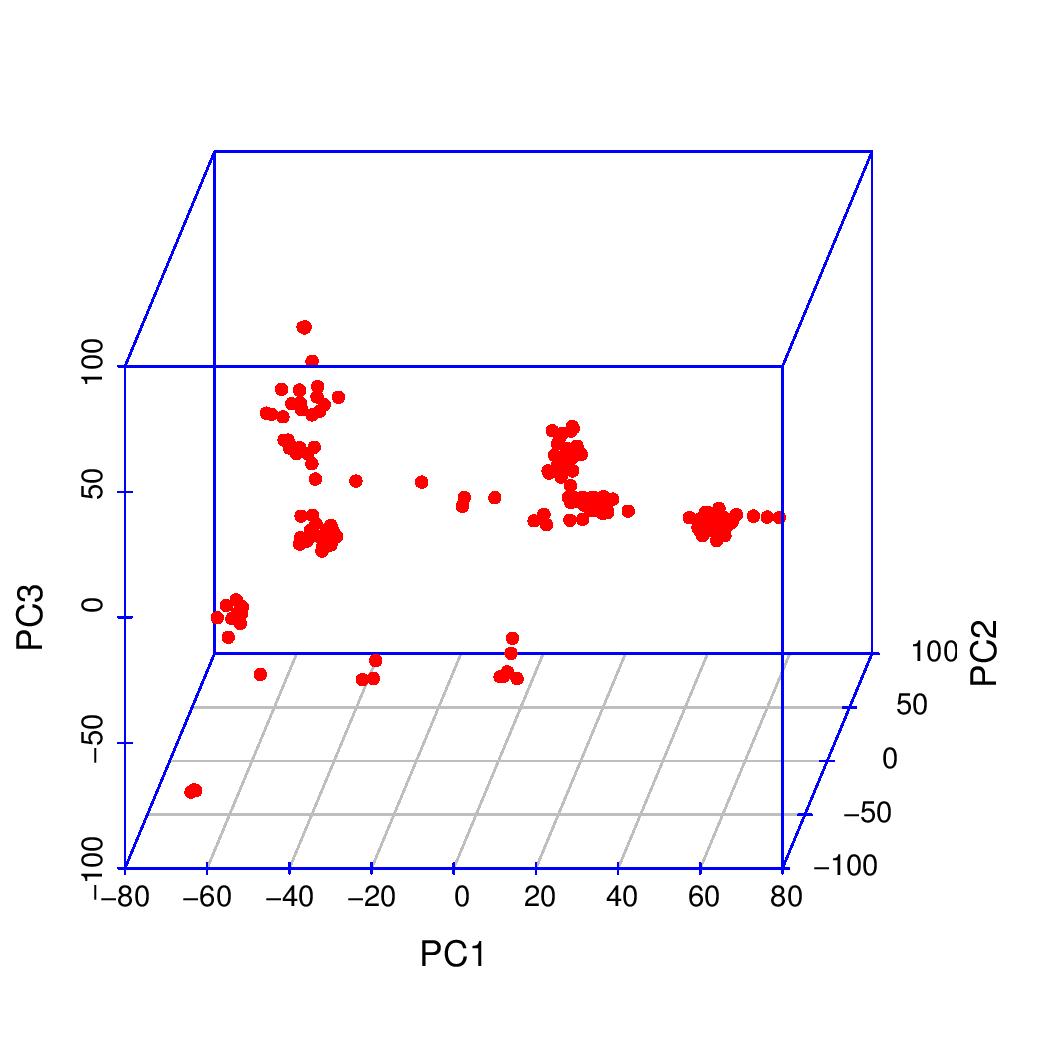


**Supplementary Fig. S1** Three-dimensional PCA scatter plot of 188 walnut genotypes


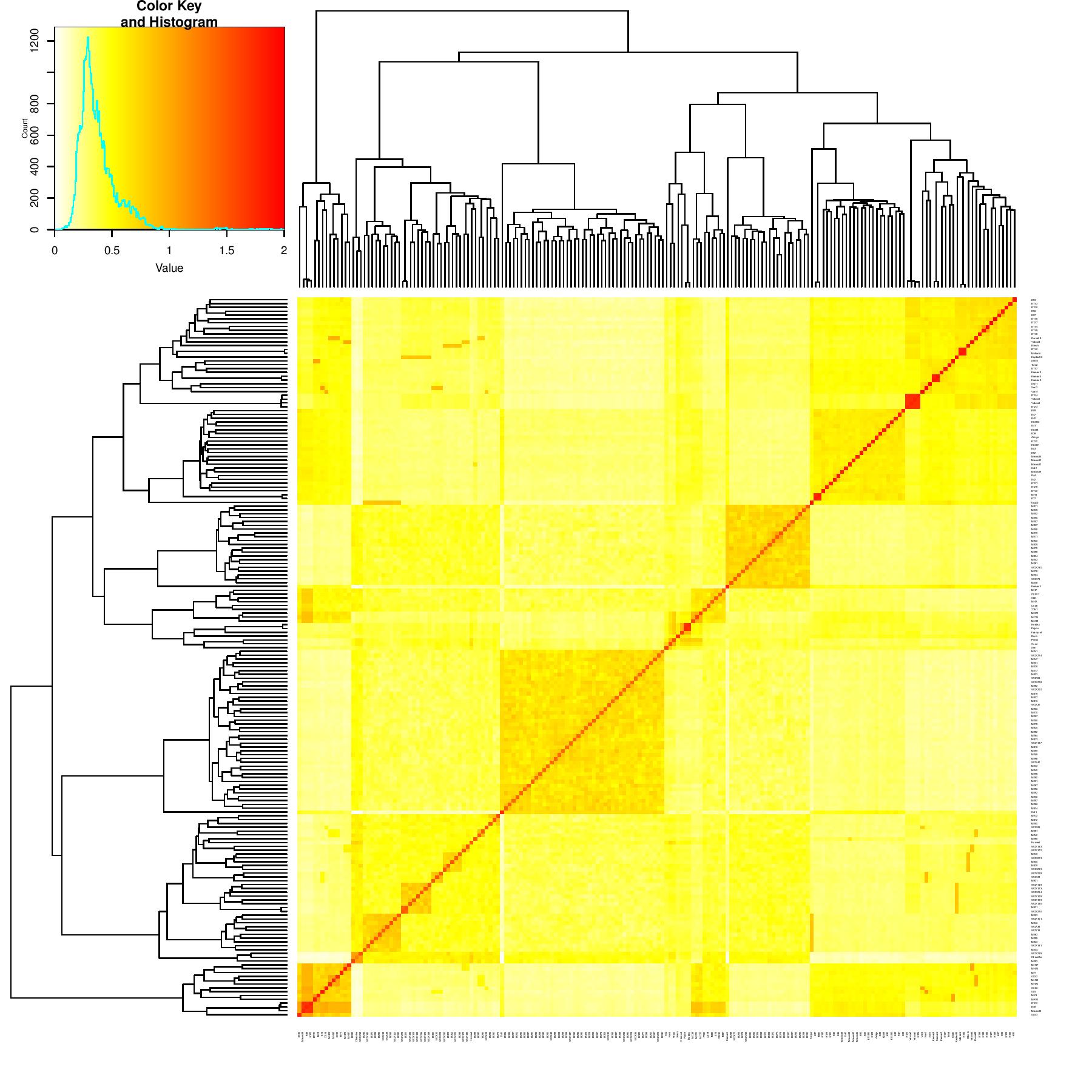


**Supplementary Fig. S2** Kinship relationship among 188 walnut genotypes


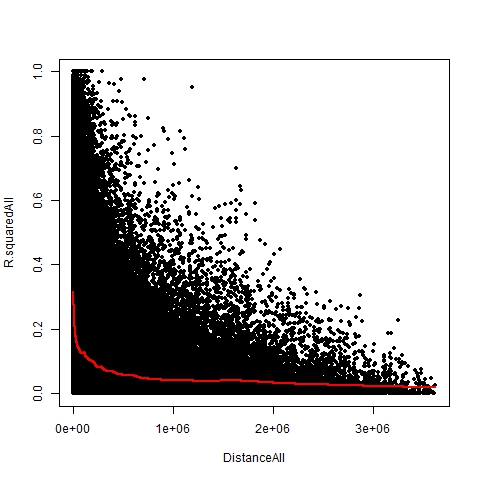


**Supplementary Fig. S3** LD decay for all SNP’s within scaffolds. The x-axis represents the distance (in bp) between each pair of SNPs and the y-axis represents the the *R^2^* value between both SNPs.
