# Supplementary material for: Major QTL with pleiotropic effects controlling time of leaf budburst and flowering-related traits in walnut (Juglans regia L.)
Source: Sci Rep. 2020 Sep 16;10:15207. doi: 10.1038/s41598-020-71809-x (PMC7495441; doi:10.1038/s41598-020-71809-x)
Supplement: Supplementary file 4 — Supplementary Tables. [file 41598_2020_71809_MOESM4_ESM.docx]

Supplementary Tables

**Major QTL with pleiotropic effects controlling time of leaf budburst and flowering-related traits in walnut (*Juglans regia* L.)**

Şakir Burak Bükücü, Mehmet Sütyemez, Sina Kefayati, Aibibula Paizila, Abdulqader Jighly, Salih Kafkas

**Supplementary Table S1** The list of walnut accessions used in this study.

| **Accessions** | **Origin** | **Description** |
| --- | --- | --- |
| Serr | USA | PI 159568 x Payne |
| B52 | Turkey | Open pollinated |
| M304 | Turkey | Open pollinated |
| KSU11 | Turkey | Open pollinated |
| WCK32 | Turkey | Open pollinated |
| M246 | Turkey | Open pollinated |
| M286 | Turkey | Open pollinated |
| M258 | Turkey | Open pollinated |
| Midland | USA | Franquette x Payne |
| Maras12 | Turkey | Open pollinated |
| C6S11 | Turkey | Open pollinated |
| M296 | Turkey | Open pollinated |
| B63 | Turkey | Open pollinated |
| KSU12 | Turkey | Open pollinated |
| M239 | Turkey | Open pollinated |
| B116 | Turkey | Open pollinated |
| B112 | Turkey | Open pollinated |
| WCK136 | Turkey | Open pollinated |
| M278 | Turkey | Open pollinated |
| B41 | Turkey | Open pollinated |
| B125 | Turkey | Open pollinated |
| B47 | Turkey | Open pollinated |
| Payne | USA | Open pollinated |
| B113 | Turkey | Open pollinated |
| WCK40 | Turkey | Open pollinated |
| Maras18 | Turkey | Open pollinated |
| M318 | Turkey | Open pollinated |
| B42 | Turkey | Open pollinated |
| B56 | Turkey | Open pollinated |
| 6S1 | Turkey | Open pollinated |
| WCK73 | Turkey | Open pollinated |
| WCK224 | Turkey | Open pollinated |
| WCK215 | Turkey | Open pollinated |
| B38 | Turkey | Open pollinated |
| WCK105 | Turkey | Open pollinated |
| WCK223 | Turkey | Open pollinated |
| MP21 | Turkey | Open pollinated |
| B127 | Turkey | Open pollinated |
| Back | Unknown | Unknown |
| Yalova3 | Turkey | Open pollinated |
| M276 | Turkey | Open pollinated |
| M297 | Turkey | Open pollinated |
| M298 | Turkey | Open pollinated |
| Howard | USA | Pedro x 56-224 |
| MC19 | Turkey | Open pollinated |
| B40 | Turkey | Open pollinated |
| M264 | Turkey | Open pollinated |
| WCK123 | Turkey | Open pollinated |
| MH26 | Turkey | Open pollinated |
| Bursa95 | Turkey | Open pollinated |
| M269 | Turkey | Open pollinated |
| WCK109 | Turkey | Open pollinated |
| WCK225 | Turkey | Open pollinated |
| Kaman1 | Turkey | Open pollinated |
| M326 | Turkey | Open pollinated |
| WCK133 | Turkey | Open pollinated |
| Yalova4 | Turkey | Open pollinated |
| M324 | Turkey | Open pollinated |
| WCK233 | Turkey | Open pollinated |
| M268 | Turkey | Open pollinated |
| M303 | Turkey | Open pollinated |
| Tokat1 | Turkey | Open pollinated |
| Franquet | France | Open pollinated |
| MP3 | Turkey | Open pollinated |
| Maras19 | Turkey | Open pollinated |
| B57 | Turkey | Open pollinated |
| M312 | Turkey | Open pollinated |
| WCK33 | Turkey | Open pollinated |
| M316 | Turkey | Open pollinated |
| M240 | Turkey | Open pollinated |
| B65 | Turkey | Open pollinated |
| B53 | Turkey | Open pollinated |
| Chandler | USA | 56-224 x Pedro |
| M327 | Turkey | Open pollinated |
| M252 | Turkey | Open pollinated |
| B115 | Turkey | Open pollinated |
| M306 | Turkey | Open pollinated |
| MC22 | Turkey | Open pollinated |
| M262 | Turkey | Open pollinated |
| B121 | Turkey | Open pollinated |
| Kaplan86 | Turkey | Open pollinated |
| M280 | Turkey | Open pollinated |
| M255 | Turkey | Open pollinated |
| 77H1 | Turkey | Open pollinated |
| M300 | Turkey | Open pollinated |
| WCK172 | Turkey | Open pollinated |
| Kaman5 | Turkey | Open pollinated |
| MH10 | Turkey | Open pollinated |
| Amigo | USA | Sharka x Marchetti |
| M267 | Turkey | Open pollinated |
| M281 | Turkey | Open pollinated |
| M263 | Turkey | Open pollinated |
| MC21 | Turkey | Open pollinated |
| Rondedemontignac | France | Open pollinated |
| M238 | Turkey | Open pollinated |
| M299 | Turkey | Open pollinated |
| WCK137 | Turkey | Open pollinated |
| M244 | Turkey | Open pollinated |
| M287 | Turkey | Open pollinated |
| M323 | Turkey | Open pollinated |
| B117 | Turkey | Open pollinated |
| M284 | Turkey | Open pollinated |
| Hartley | USA | Open pollinated |
| 6S13 | Turkey | Open pollinated |
| 6S12 | Turkey | Open pollinated |
| MH2 | Turkey | Open pollinated |
| MH7 | Turkey | Open pollinated |
| Kaman3 | Turkey | Open pollinated |
| M253 | Turkey | Open pollinated |
| M329 | Turkey | Open pollinated |
| Urgup | Turkey | Open pollinated |
| B114 | Turkey | Open pollinated |
| WCK35 | Turkey | Open pollinated |
| M307 | Turkey | Open pollinated |
| Kaman4 | Turkey | Open pollinated |
| M289 | Turkey | Open pollinated |
| M311 | Turkey | Open pollinated |
| Sen1 | Turkey | Open pollinated |
| M275 | Turkey | Open pollinated |
| KSU5 | Turkey | Open pollinated |
| WCK200 | Turkey | Open pollinated |
| M266 | Turkey | Open pollinated |
| M315 | Turkey | Open pollinated |
| M260 | Turkey | Open pollinated |
| M279 | Turkey | Open pollinated |
| M272 | Turkey | Open pollinated |
| WCK234 | Turkey | Open pollinated |
| M256 | Turkey | Open pollinated |
| Sebin | Turkey | Open pollinated |
| M241 | Turkey | Open pollinated |
| B122 | Turkey | Open pollinated |
| Bilecik | Turkey | Open pollinated |
| Maras14 | Turkey | Open pollinated |
| B120 | Turkey | Open pollinated |
| M305 | Turkey | Open pollinated |
| B126 | Turkey | Open pollinated |
| M242 | Turkey | Open pollinated |
| B119 | Turkey | Open pollinated |
| M249 | Turkey | Open pollinated |
| M270 | Turkey | Open pollinated |
| WCK65 | Turkey | Open pollinated |
| WCK228 | Turkey | Open pollinated |
| Maras10 | Turkey | Open pollinated |
| M288 | Turkey | Open pollinated |
| M291 | Turkey | Open pollinated |
| Van4 | Turkey | Open pollinated |
| M261 | Turkey | Open pollinated |
| M292 | Turkey | Open pollinated |
| M301 | Turkey | Open pollinated |
| WCK141 | Turkey | Open pollinated |
| C6S4 | Turkey | Open pollinated |
| M271 | Turkey | Open pollinated |
| Sut2 | Turkey | Open pollinated |
| B44 | Turkey | Open pollinated |
| M293 | Turkey | Open pollinated |
| M247 | Turkey | Open pollinated |
| MH20 | Turkey | Open pollinated |
| B124 | Turkey | Open pollinated |
| B49 | Turkey | Open pollinated |
| MH17 | Turkey | Open pollinated |
| WCK101 | Turkey | Open pollinated |
| M313 | Turkey | Open pollinated |
| M248 | Turkey | Open pollinated |
| Pedro | USA | Conway Mayette x Payne |
| Sen2 | Turkey | Open pollinated |
| MH1 | Turkey | Open pollinated |
| WCK119 | Turkey | Open pollinated |
| WCK15 | Turkey | Open pollinated |
| M320 | Turkey | Open pollinated |
| B123 | Turkey | Open pollinated |
| M290 | Turkey | Open pollinated |
| WCK84 | Turkey | Open pollinated |
| Sutyemez1 | Turkey | Open pollinated |
| 6S6 | Turkey | Open pollinated |
| M282 | Turkey | Open pollinated |
| WCK210 | Turkey | Open pollinated |
| M257 | Turkey | Open pollinated |
| C6S9 | Turkey | Open pollinated |
| M243 | Turkey | Open pollinated |
| M277 | Turkey | Open pollinated |
| M321 | Turkey | Open pollinated |
| MF1 | Turkey | Open pollinated |
| WCK226 | Turkey | Open pollinated |
| Yalova1 | Turkey | Open pollinated |
| B118 | Turkey | Open pollinated |
| B37 | Turkey | Open pollinated |
| M251 | Turkey | Open pollinated |
| M283 | Turkey | Open pollinated |

**Supplementary Table S2** Code, units, maximum, minimum, mean, standard deviation, and h2 values of 13 phonological traits in the walnut accessions

| **Traits** | **Code** | **Units** | **Year** | **N** | **Min** | **Max** | **Mean** | **Std. Deviaiton** | **h^2^ _(narrow sense heritability)_** |
| --- | --- | --- | --- | --- | --- | --- | --- | --- | --- |
| Date of bud burst | T01 | Julian date | 2016 | 188 | 72 | 105 | 87.27 | 5.776 | 0.720 |
| Date of bud burst | T01 | Julian date | 2017 | 188 | 73 | 109 | 89.37 | 6.175 | 0.829 |
| Date of bud burst | T01 | Julian date | 2018 | 188 | 67 | 102 | 83.84 | 7.125 | 0.853 |
| Date of bud burst | T01 | Julian date | Mean | 188 | 71 | 105 | 86.80 | 6.050 | 0.877 |
| Leafing date | T02 | Julian date | 2016 | 188 | 83 | 111 | 96.27 | 4.355 | 0.779 |
| Leafing date | T02 | Julian date | 2017 | 188 | 86 | 117 | 100.96 | 6.436 | 0.832 |
| Leafing date | T02 | Julian date | 2018 | 188 | 76 | 107 | 92.53 | 6.586 | 0.930 |
| Leafing date | T02 | Julian date | Mean | 188 | 82 | 112 | 96.56 | 5.612 | 0.888 |
| First female bloom date | T03 | Julian date | 2016 | 188 | 92 | 121 | 104.74 | 5.423 | 0.850 |
| First female bloom date | T03 | Julian date | 2017 | 188 | 84 | 124 | 110.00 | 5.880 | 0.904 |
| First female bloom date | T03 | Julian date | 2018 | 188 | 83 | 118 | 100.22 | 6.639 | 0.885 |
| First female bloom date | T03 | Julian date | Mean | 188 | 90 | 121 | 104.96 | 5.560 | 0.936 |
| First male bloom date | T04 | Julian date | 2016 | 95 | 92 | 112 | 101.09 | 5.860 | 0.861 |
| First male bloom date | T04 | Julian date | 2017 | 150 | 93 | 119 | 106.76 | 7.371 | 0.894 |
| First male bloom date | T04 | Julian date | 2018 | 184 | 84 | 116 | 97.97 | 6.998 | 0.806 |
| First male bloom date | T04 | Julian date | Mean | 185 | 90 | 117 | 102.05 | 6.679 | 0.715 |
| Last female bloom date | T05 | Julian date | 2016 | 188 | 99 | 137 | 115.42 | 6.594 | 0.662 |
| Last female bloom date | T05 | Julian date | 2017 | 188 | 107 | 140 | 123.57 | 5.802 | 0.786 |
| Last female bloom date | T05 | Julian date | 2018 | 188 | 97 | 137 | 113.59 | 8.438 | 0.748 |
| Last female bloom date | T05 | Julian date | Mean | 188 | 101 | 138 | 117.51 | 6.494 | 0.846 |
| Last male bloom date | T06 | Julian date | 2016 | 95 | 98 | 124 | 107.89 | 5.915 | 0.574 |
| Last male bloom date | T06 | Julian date | 2017 | 150 | 100 | 126 | 114.23 | 5.732 | 0.954 |
| Last male bloom date | T06 | Julian date | 2018 | 184 | 94 | 124 | 105.58 | 7.047 | 0.807 |
| Last male bloom date | T06 | Julian date | Mean | 185 | 97 | 124 | 109.03 | 6.293 | 0.611 |
| Female flowering period | T07 | Julian date | 2016 | 188 | 4 | 22 | 10.62 | 3.377 | 0.389 |
| Female flowering period | T07 | Julian date | 2017 | 188 | 8 | 20 | 13.35 | 2.544 | 0.000 |
| Female flowering period | T07 | Julian date | 2018 | 188 | 6 | 25 | 13.35 | 3.548 | 0.000 |
| Female flowering period | T07 | Julian date | Mean | 188 | 7 | 19 | 12.44 | 2.209 | 0.123 |
| Male flowering period | T08 | Julian date | 2016 | 95 | 2 | 14 | 6.79 | 2.505 | 0.047 |
| Male flowering period | T08 | Julian date | 2017 | 150 | 2 | 15 | 7.47 | 3.191 | 0.549 |
| Male flowering period | T08 | Julian date | 2018 | 184 | 2 | 15 | 7.64 | 2.450 | 0.368 |
| Male flowering period | T08 | Julian date | Mean | 185 | 2 | 13 | 7.12 | 2.136 | 0.136 |
| Catkin Abundance | T09 | Scale | 2016 | 95 | 3 | 7 | 5.15 | 1.376 | 0.203 |
| Catkin Abundance | T09 | Scale | 2017 | 150 | 3 | 7 | 4.87 | 1.441 | 0.363 |
| Catkin Abundance | T09 | Scale | 2018 | 184 | 3 | 7 | 4.84 | 1.462 | 0.186 |
| Catkin Abundance | T09 | Scale | Mean | 185 | 3 | 7 | 4.78 | 1.366 | 0.188 |
| Female Abundance | T10 | Scale | 2016 | 188 | 3 | 7 | 5.55 | 1.400 | 0.297 |
| Female Abundance | T10 | Scale | 2017 | 188 | 3 | 7 | 5.55 | 1.639 | 0.365 |
| Female Abundance | T10 | Scale | 2018 | 188 | 3 | 7 | 5.65 | 1.457 | 0.439 |
| Female Abundance | T10 | Scale | Mean | 188 | 3 | 7 | 5.65 | 1.397 | 0.455 |
| Nut setting type | T11 | Scale | 2016 | 188 | 1 | 4 | 2.13 | 0.412 | 0.058 |
| Nut setting type | T11 | Scale | 2017 | 188 | 2 | 4 | 2.22 | 0.443 | 0.242 |
| Nut setting type | T11 | Scale | 2018 | 188 | 2 | 4 | 2.22 | 0.439 | 0.196 |
| Nut setting type | T11 | Scale | Mean | 188 | 2 | 4 | 2.21 | 0.420 | 0.147 |
| Inflorescence habit | T12 | Scale | 2016 | 95 | 1 | 3 | 1.64 | 0.798 | 0.018 |
| Inflorescence habit | T12 | Scale | 2017 | 150 | 1 | 3 | 1.75 | 0.914 | 0.601 |
| Inflorescence habit | T12 | Scale | 2018 | 184 | 1 | 3 | 1.61 | 0.802 | 0.585 |
| Inflorescence habit | T12 | Scale | Mean | 185 | 1 | 3 | 1.62 | 0.813 | 0.552 |
| Lateral bud flowering | T13 | % | 2016 | 188 | 0 | 100 | 65.90 | 28.467 | 0.446 |
| Lateral bud flowering | T13 | % | 2017 | 188 | 0 | 100 | 60.41 | 37.136 | 0.465 |
| Lateral bud flowering | T13 | % | 2018 | 188 | 0 | 100 | 67.85 | 30.903 | 0.453 |
| Lateral bud flowering | T13 | % | Mean | 188 | 3 | 100 | 64.70 | 28.429 | 0.538 |
